# Dynamic Network Connectivity Analysis to Identify Epileptogenic Zones Based on Stereo-Electroencephalography

**DOI:** 10.3389/fncom.2016.00113

**Published:** 2016-10-27

**Authors:** Jun-Wei Mao, Xiao-Lai Ye, Yong-Hua Li, Pei-Ji Liang, Ji-Wen Xu, Pu-Ming Zhang

**Affiliations:** ^1^School of Biomedical Engineering, Shanghai Jiao Tong UniversityShanghai, China; ^2^Department of Functional Neurosurgery, Renji Hospital, School of Medicine, Shanghai Jiao Tong UniversityShanghai, China

**Keywords:** stereo-EEG, epileptogenic zones, Kalman filter, time-variant partial directed coherence, graph theory

## Abstract

**Objectives:** Accurate localization of epileptogenic zones (EZs) is essential for successful surgical treatment of refractory focal epilepsy. The aim of the present study is to investigate whether a dynamic network connectivity analysis based on stereo-electroencephalography (SEEG) signals is effective in localizing EZs.

**Methods:** SEEG data were recorded from seven patients who underwent presurgical evaluation for the treatment of refractory focal epilepsy and for whom the subsequent resective surgery gave a good outcome. A time-variant multivariate autoregressive model was constructed using a Kalman filter, and the time-variant partial directed coherence was computed. This was then used to construct a dynamic directed network model of the epileptic brain. Three graph measures (in-degree, out-degree, and betweenness centrality) were used to analyze the characteristics of the dynamic network and to find the important nodes in it.

**Results:** In all seven patients, the indicative EZs localized by the in-degree and the betweenness centrality were highly consistent with the clinically diagnosed EZs. However, the out-degree did not indicate any significant differences between nodes in the network.

**Conclusions:** In this work, a method based on ictal SEEG signals and effective connectivity analysis localized EZs accurately. The results suggest that the in-degree and betweenness centrality may be better network characteristics to localize EZs than the out-degree.

## 1. Introduction

Focal epilepsy, in which the origin of epileptic seizures is limited to one hemisphere (Berg et al., 2010), is common and comprising more than 50% of patients with epilepsy (Hauser et al., 1996). Despite great developments in pharmacological treatment, about 30–50% of patients with focal epilepsy cannot be sufficiently controlled with antiepileptic drugs (Beleza, 2009). For these patients, surgical resection of the epileptogenic zones (EZs), the brain areas that are essential for the generation of epileptic seizures (Rosenow and Luders, 2001), may be the only way to suppress or reduce seizures.

EZs can sometimes be adequately identified by a combination of non-invasive techniques, such as analysis of ictal symptomatology, neurological examination, electroencephalography (EEG), magnetoencephalography (MEG), and magnetic resonance imaging (MRI) (Rosenow and Luders, 2001). However, when the EZs cannot be precisely identified, invasive intracranial EEG recordings are required. Stereo-EEG (SEEG) can record neural activities by stereotactic placement of intracranial electrodes within different brain regions, especially in deep areas (Bancaud and Talairach, 1973). In association with long-term video recordings, SEEG allows planning of tailored resections based on individual anatomical and electro-clinical characteristics (Varotto et al., 2012). SEEG is a relatively safe tool and has been considered the gold standard for EZ identification (Cossu et al., 2005).

In clinical practice, EZs are usually identified by visual inspection of ictal SEEG signals. Experienced neurologists can analyze the amplitude, frequency, and relation between signals from each recording channel to identify EZs. However, this is time-consuming and inevitably affected by subjective factors. Moreover, the rather high failure rate of surgical treatment of extratemporal epilepsies (Téllez-Zenteno et al., 2005) underlines that the precise identification of EZs is still an unsolved problem and requires more sophisticated methods to mine further information from SEEG signals.

In recent decades, many computational methods have been proposed to investigate SEEG signals, among which effective connectivity analysis is one of the most widely used. The effective connectivity is defined as the influence that one neuronal system exerts over another, either directly or indirectly (Friston et al., 1993). It is based on Granger causality (Granger, 1969) derived from multivariate autoregressive (MVAR) modeling of multichannel EEG signals and has been successfully used to study the flow of seizures in patients with focal epilepsy (Franaszczuk et al., 1994; Franaszczuk and Bergey, 1998; Varotto et al., 2012). One effective tool to evaluate the effective connectivity is the partial directed coherence (PDC), a frequency-domain measure based on the MVAR coefficients (Baccalá and Sameshima, 2001). In contrast to the directed transfer function (DTF), which is another frequency-domain measure and has also been widely used to study the flow of seizures (Kaminski and Blinowska, 1991; Franaszczuk et al., 1994; Franaszczuk and Bergey, 1998; Ding et al., 2007; Lu et al., 2012; Kim et al., 2014; Basu et al., 2015), PDC can distinguish indirect and direct causalities, the latter being important for localizing EZs. However, both PDC and DTF are based on the MVAR model and are limited to stationary signals. To overcome this limitation, the time-variant MVAR (TVMVAR) model has been introduced (Astolfi et al., 2008; Wilke et al., 2008), and time-variant seizures have been successfully studied using PDC and DTF based on this model, i.e., time-variant PDC (TVPDC) and time-variant DTF (TVDTF) (Wilke et al., 2008, 2009; Leistritz et al., 2013; van Mierlo et al., 2013; Coito et al., 2015). The TVMVAR model is usually estimated using a Kalman filter (Arnold et al., 1998; Schlogl et al., 2000; Milde et al., 2010). As the Kalman filter estimates the system states related to the TVMVAR model's coefficients iteratively, it takes a period of data to follow the real states during the initial estimation procedure (i.e., the adaptation process). Thus, the states estimated during the adaptation process are incorrect and should be discarded (van Mierlo et al., 2013). Actually, the adaptation determines how quickly the Kalman filter can precisely follow the system's dynamics, and an incorrect estimation of the adaptation time will lead to an incorrect estimation of states. In this paper, adaptation is quantitatively evaluated to ensure correct estimation of the time-variant connectivity during propagation of seizures.

Both DTF and PDC measure the information flow from one channel to another for all possible pairs in a network. However, the evaluation of interconnections between all these pairs in a particular frequency band will produce huge matrices of correlation data, which makes a statistical interpretation difficult (Panzica et al., 2013). Graph theory is a promising mathematical framework that is widely used to quantify topological properties of complex networks (Boccaletti et al., 2006), especially the epileptic neural network (Chiang and Haneef, 2014; Haneef and Chiang, 2014). Wilke et al. (2011) found that the betweenness centrality was correlated with the location of resected cortical regions in patients who were seizure-free following surgical intervention. Varotto et al. (2012) analyzed the connectivity pattern in the brain networks of patients with type II focal cortical dysplasia and found that the lesional nodes played a leading role in generating and propagating ictal activities in EEG recordings by acting as the hubs of the epileptic network. van Mierlo et al. (2013) found that the electrode contact with the highest out-degree always lay within the resected brain regions and that the patient-specific connectivity patterns were consistent over the majority of seizures. Sethi et al. (2016) analyzed a network constructed from functional MRI (fMRI) data from patients with polymicrogyria and refractory epilepsy, and found that the polymicrogyric nodes showed significantly increased clustering coefficients and characteristic path lengths compared with the normal contralateral homologous cortical regions. Thus, graph theory is an effective method to identify the highly interconnected hubs in the epileptic network, which is helpful in localizing the EZs in the network. As different graph indices define different types of hubs, the effectiveness of each graph index should be evaluated with a clinical dataset.

In this paper, TVPDC and graph theory are applied to analyze the SEEG signals of patients with refractory focal epilepsy to identify EZs. Three graph indices, namely, in-degree, out-degree, and betweenness centrality, are used to characterize the network dynamics during ictal onset and early propagation. The brain areas where the electrode contacts with relatively high index values are located are considered as the indicative EZs. These indicative EZs are compared with the conclusions from visual analyses of SEEG signals performed by epileptologists, and the effectiveness of each graph index is evaluated. It is found that the in-degree and the betweenness centrality are effective in localizing EZs, but the out-degree is not effective. As this framework requires little prior knowledge other than the seizure onset time, it may provide epileptologists with some objective guidance regarding the abnormal regions.

## 2. Materials and methods

### 2.1. Time-variant effective connectivity

The TVMVAR model is a parametric model that represents a multivariate dataset as a time-variant linear combination of previous samples plus white noise. It is usually used to describe non-stationary process and is characterized by

(1) $$X(n)=\sum_{r\;=\;1}^{p}A_{r}(n)X(n-r)\;+\;E(n)$$

where $X\left(n\right)={\left[x_{1}\left(n\right),x_{2}\left(n\right),\ldots ,x_{d}\left(n\right)\right]}^{T}$ is the data vector at time *n*, *d* is the number of recording channels, *p* is the model order, *A*_*r*_(*n*) is a time-dependent *d*×*d* matrix of model coefficients for time delay *r*, and $E\left(n\right)={\left[e_{1}\left(n\right),e_{2}\left(n\right),\ldots ,e_{d}\left(n\right)\right]}^{T}$ is a vector of white noise at time *n*.

The Kalman filter algorithm is an efficient method to estimate the coefficients of the TVMVAR model (Arnold et al., 1998; Schlogl et al., 2000; Milde et al., 2010). It takes the coefficients of the TVMVAR model as the state of a state model

(2) $$S_{n}=S_{n\;-\;1}+W_{n}$$

and the TVMVAR model is rewritten as a measurement equation

(3) $$O_{n}=H_{n}S_{n}+V_{n}$$

where *O*_*n*_, *S*_*n*_, and *H*_*n*_ connect variables of the TVMVAR model and are given by

(4) $$S_{n}=\left[\begin{array}{c} A_{1}{\left(n\right)}^{T}\\ \vdots \\ A_{p}{\left(n\right)}^{T} \end{array}\right]$$

(5) $$O_{n}=\left[\begin{array}{c} x_{1}(n)\ldots x_{d}(n) \end{array}\right]=X^{T}(n)$$

(6) $$H_{n}=\left[\begin{array}{c} O_{n\;-\;1}\ldots O_{n\;-\;p} \end{array}\right]$$

As can be seen, Equation (2) models the state process *S*_*n*_ as a random process with an additive noise *W*_*n*_, and Equation (3) connects the state process with the observation via a transition matrix *H*_*n*_ and another additive noise *V*_*n*_. Then the classical computational steps of a Kalman filter are used to estimate *S*_*n*_, which consists of the TVMVAR coefficients as shown in Equation (4). The initial state for the first iteration is defined as *S*_*p*_ = 0, and the prediction error covariance as *P*_*p*_ = *I*_*dp*_ (the identity matrix of size *d*×*p*), and the following steps are then repeated for each recoding sample:

(7) $${\hat{S}}_{n}^{-}={\hat{S}}_{n\;-\;1}$$

(8) $$P_{n}^{-}=P_{n\;-\;1}+\bar{W_{n}},\text{ where }\bar{W_{n}}=EW_{n}W_{n}^{T}$$

(9) $$K_{n}=P_{n}^{-}H_{n}^{T}{\left(H_{n}P_{n}^{-}H_{n}^{T}+\bar{V_{n}}\right)}^{-1},\text{where }\bar{V_{n}}=EV_{n}V_{n}^{T}$$

(10) $${\hat{S}}_{n}={\hat{S}}_{n}^{-}+K_{n}(O_{n}-H_{n}{\hat{S}}_{n}^{-})$$

(11) $$P_{n}=(I_{dp}-K_{n}H_{n})P_{n}^{-}$$

where *E* denotes the expectation, ^^^ denotes the a *posteriori* estimate, and ^−^ denotes the a *priori* estimate. The covariance matrices *V*_*n*_ are computed recursively as follows (Milde et al., 2010):

(12) $$\bar{V_{0}}=I_{d},\;\bar{V_{n}}=\bar{V_{n\;-\;1}}(1-\lambda )+\lambda {\left(O_{n}-H_{n}S_{n}\right)}^{T}(O_{n}-H_{n}S_{n})$$

where *I*_*d*_ is the identity matrix of size *d*, and the factor λ works as an update coefficient and has to be chosen between 0 and 1. The matrix $\bar{W_{n}}$ can be chosen constantly as a weighted identity matrix $\bar{W_{n}}=\Lambda I_{dp}$ with a weighting constant Λ between 0 and 1. It is appropriate to choose identical λ and Λ (Milde et al., 2010).

In order to select a proper update coefficient (λ, equal to Λ) and determine the adaptation time of the Kalman filter, two Kalman filters with same settings but different start times of the data are used. The filter that starts with the earlier data frame should become adapted earlier, so if the filter that starts later has been adapted, the states estimated by the two filters at the same time should show little difference, and the adaptation time is then defined as the time period that the filter that starts later has taken to become adapted. Here, the difference of the model coefficients is measured using the relative squared error (RSE):

(13) $$\begin{array}{l} \text{RSE}(n)\\ =\frac{\sum {\left(\text{vec}([A_{1}(n),\ldots ,A_{p}(n)])-\text{vec}([B_{1}(n),\ldots ,B_{p}(n)])\right)}^{2}}{\sum {\left(|\text{vec}([A_{1}(n),\ldots ,A_{p}(n)])|+|\text{vec}([B_{1}(n),\ldots ,B_{p}(n)])|\right)}^{2}} \end{array}$$

where *A* and *B* are the respective matrices of the model coefficients estimated by the two Kalman filters, and *vec* refers to converting the matrix to a vector. As the adaptation time is defined by the Kalman filter that starts later, the lag in start time does not matter.

As described above, a set of time-dependent coefficients in the TVMVAR model are estimated. In order to examine the causal relation between signals from different channels in the spectral domain, the Fourier transform of Equation (1) is calculated as follows:

(14) A(f,n)X(f,n) = E(f,n)

where

(15) $$A(f,n)=-\sum_{r\;=\;0}^{p}A_{r}(n)e^{-i2\pi fr}$$

with *A*_0_(*n*) = −*I*_*d*_ (the identity matrix of size *d*); *A*(*f, n*), *X*(*f, n*), and *E*(*f, n*) are the Fourier transforms of the coefficient matrices *A*_*r*_(*n*), the time series *X*(*n*), and the residuals *E*(*n*), respectively.

Then a spectrum-weighted PDC (swPDC) from channel *j* to *i* in a frequency band (*f*_1_, *f*_2_) is calculated as follows (Astolfi et al., 2006; Plomp et al., 2014):

(16) $${\text{swPDC}}_{ij}(n)=\;\frac{\sum_{f\;=\;f_{1}}^{f_{2}}{\text{sPDC}}_{ij}(f,n)S_{j}(f,n)}{\sum_{f\;=\;f_{1}}^{f_{2}}S_{j}(f,n)}$$

where the lower frequency bound *f*_1_ is used to remove background electrical activity, and the upper frequency bound *f*_2_ is usually limited by the resampling rate. In this study, *f*_1_ and *f*_2_ are set to be 3 and 40 Hz, respectively, which covers the majority of the power in SEEG signals around seizure onset (van Mierlo et al., 2013). *S*_*j*_(*f, n*) is the time-variant power spectrum at the source region of *j* calculated by the short-time Fourier transform (STFT). The sPDC is calculated as follows (Astolfi et al., 2006):

(17) $${\text{sPDC}}_{ij}(f,n)=\;\frac{|A_{ij}(f,n){|}^{2}}{\sum_{k\;=\;1}^{d}|A_{kj}(f,n){|}^{2}}$$

where *A*_*ij*_(*f, n*) is the element in the *i*th row and *j*th column of the matrix *A*(*f, n*).

### 2.2. Graph analysis

A graph is an abstract of a network consisting of a set of nodes (vertices) and edges. In our study, nodes represent brain regions at which the electrode contacts were located, edges denote interactions between these regions, and the strength of the connectivity between nodes is represented by the swPDC value, which ranges from 0 to 1.

Graphs can be characterized by various measures. One of the simplest quantitative indices of a node is its degree, which indicates the sum of weighted connections from (in-degree deg_in_) or toward (out-degree deg_out_) all of the other nodes, and represents the most common measure of centrality.

As the number of network nodes differs among patients, the in-degree and out-degree of each node are normalized by the number of network nodes *d* as follows (Varotto et al., 2012):

(18) $${\text{deg}}_{\text{in}}(i)=\;\frac{\sum_{j\;\neq \;i}{\text{swPDC}}_{ij}}{d}$$

(19) $${\text{deg}}_{\text{out}}(j)=\;\frac{\sum_{i\;\neq \;j}{\text{swPDC}}_{ij}}{d}$$

Another measure of centrality is the betweenness centrality (*bc*), defined as the ratio of the number of the shortest paths that pass through a specified node *v* to the total number of the shortest paths in the network (Wang et al., 2008):

(20) $$\text{bc}(v)=\sum_{i\;\neq \;j\;\neq \;v\in V}\frac{\sigma_{ij}(v)}{\sigma_{ij}}$$

where σ_*ij*_ is the number of shortest paths between nodes *i* and *j*, and σ_*ij*_(*v*) is the number of these shortest paths that pass through node *v*. The betweenness centrality is a measure of the “importance” of each node to information transmission in the network. The nodes that have a relatively high betweenness centrality act as “hubs” in a network, and removal of these nodes will change the network performance significantly (Wilke et al., 2011).

### 2.3. Patient data

The SEEG dataset was obtained from seven patients who underwent presurgical evaluation at the Department of Functional Neurosurgery in Renji Hospital (Shanghai, China). The patients included in the study were selected based on the following criteria: focal ictal onset and subsequent resective surgery giving good outcome (with the seizure frequency reduced by at least 50%, and the seizure severity also reduced significantly) during a minimum follow-up of 8 months. The study was approved by the Ethics Committee of Renji Hospital, School of Medicine, Shanghai Jiao Tong University, and all patients gave written informed consent that their clinical data might be used for research purposes. The characteristics of the patients are described in Table 1.

**Table 1:** **Clinical patient characteristics**.

**Patient**	**Gender**	**Ages (first seizure/surgery)**	**MRI findings**	**Outcome (%)**	**Postoperative follow-up (months)**
1	M	15/20	Normal	100	21
2	F	9/25	Normal	100	16
3	F	5/5.25	R, PG & PS FCD	100	20
4	F	5/34	L, PO FCD	85	14
5	F	3/19	Normal	50	16
6	M	8/28	R, SFG FCD	100	9
7	M	10/19	R, FL	80	8

*M, male; F, female; L, left; R, right; PG, precentral gyrus; PS, precentral sulcus; FCD, focal cortical dysplasia; PO, parietal–occipital; SFG, superior frontal gyrus; FL, frontal lobe*.

*The percentage in the outcome column indicates the reduction in seizure frequency, with 100% meaning that the patient was seizure-free*.

Multi-lead electrodes (HKHS Healthcare, Beijing, China; 5–18 contacts each; diameter 0.8 mm and length 2 mm, and 1.5 mm apart for each contact) were implanted stereotactically. The SEEG signals were recorded using a common reference electrode (Nikon-Kohden system; 128 channels; sampling rate 512 or 1024 Hz) under video and clinical control.

### 2.4. Identification of the epileptogenic zones

For each patient, the ictal SEEG signals around the seizure onset time, which was marked by experienced epileptologists, were selected. As the multivariate causality measures were very sensitive to the data preprocessing (Florin et al., 2010), the dataset was decimated to 128 Hz without any filtering, and its mean was set to be zero. For each resampled epoch, the order of the TVMVAR model was empirically set to be 7.

To select a proper update coefficient and determine the adaptation time of the Kalman filter, a set of update coefficients (10^−1^, 10^−2^, …, 10^−8^) were used. The update coefficient with stable and fast adaptation was selected. The time lag of the two Kalman filters was set as 20 s. Then the adaptation time was determined as the time when the RSE reached 0.5%.

Once the update coefficient was selected and the adaptation time was determined, the TVMVAR coefficient matrices from 40 s before until 40 s after the marked seizure onset time were calculated. The coefficient matrices were smoothed with a sliding window (length 0.5 s, overlap 0.125 s). Then the swPDC was calculated in the frequency band (3–40 Hz) based on Equation (16). The three graph indices (in-degree, out-degree, and betweenness centrality) were then calculated based on the swPDC. Then the results within a window from 10 s before until 5 s after the marked seizure onset time were averaged to identify the EZs.

**Figure 1:**
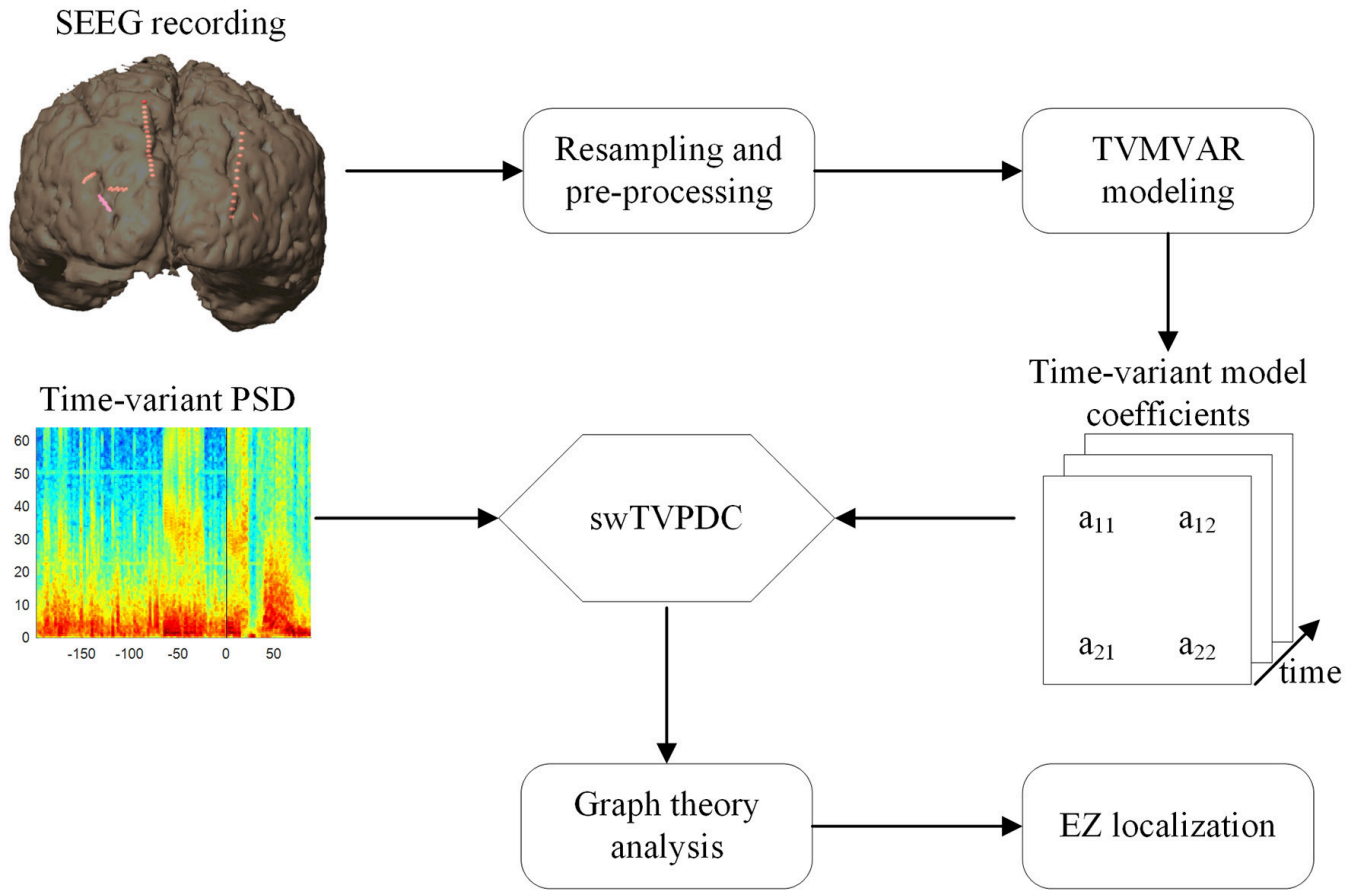
**Overview of the methods utilized in this study**.

In order to identify the EZs quantitatively, 50% of the maximum was set as the threshold (Wilke et al., 2009, 2011), and the brain areas where the electrode contacts with values exceeding this threshold were located were considered as the indicative EZs. For each graph index, we compared the identified EZs with the epileptologists' results. An overview of the methods used in this study is shown in Figure 1.

To evaluate the ability of each index used to identify EZs, we performed a receiver operating characteristic (ROC) curve analysis. Using the results of clinical diagnosis, we set the true positives to be the regions that were identified as EZs clinically and that had high graph values, and the false positives to be the regions that had high graph values but were not identified as EZs clinically. The area under the ROC curve (AUC) measures the effectiveness of the classifier, i.e., AUC ≅ 1 means that the corresponding graph index is an effective classifier to identify EZs.

## 3. Results

### 3.1. Adaptation of kalman filter

**Figure 2:**
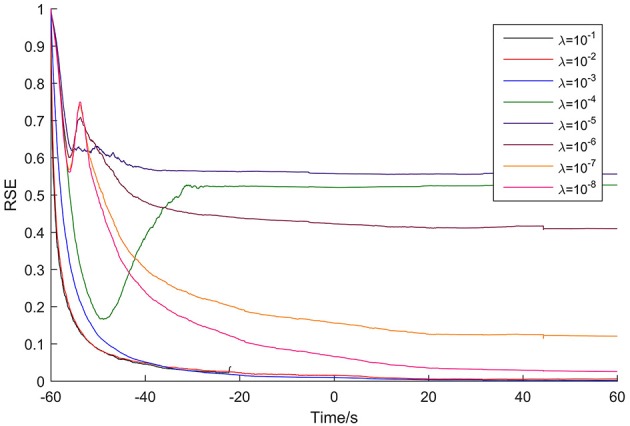
**Adaptation of the Kalman filter for Patient 1**. The optimal update coefficient is 10^−3^ (blue line).

The adaptation of the Kalman filter was evaluated for each patient to optimize the update coefficient and determine the adaptation time. As an example, Figure 2 shows the results for Patient 1, with time 0 corresponding to the seizure onset time. As the figure shows, the highest update coefficient (10^−1^, black line) leads the Kalman filter to be unstable (the line breaks at −22 s because of the appearance of the numerical value NaN). Taking account of the adaptation speed and the final RSE value, the optimal update coefficient of the Kalman filter for this patient is 10^−3^ (blue line). For the other update coefficients, the Kalman filter either adapts slowly or terminates with high RSE values.

The adaptation time was defined as the time spent until the RSE value reached 0.5%. So, for this case, the adaptation time is 73 s. The optimal update coefficient and adaptation time for the seven patients are listed in Table 2.

**Table 2:** **The optimal update coefficients and adaptation time**.

**Patient**	**Optimal update coefficient**	**Adaptation time (s)**
1	10^−3^	73
2	10^−3^	52
3	10^−3^	60
4	10^−3^	58
5	10^−3^	61
6	10^−3^	63
7	10^−3^	67

### 3.2. Case study

For each patient, the dynamic networks were constructed by the time-variant method described in Section 2. The three graph indices (in-degree, out-degree, and betweenness centrality), were then calculated to identify the important nodes in the ictal epileptic networks.

**Figure 3:**
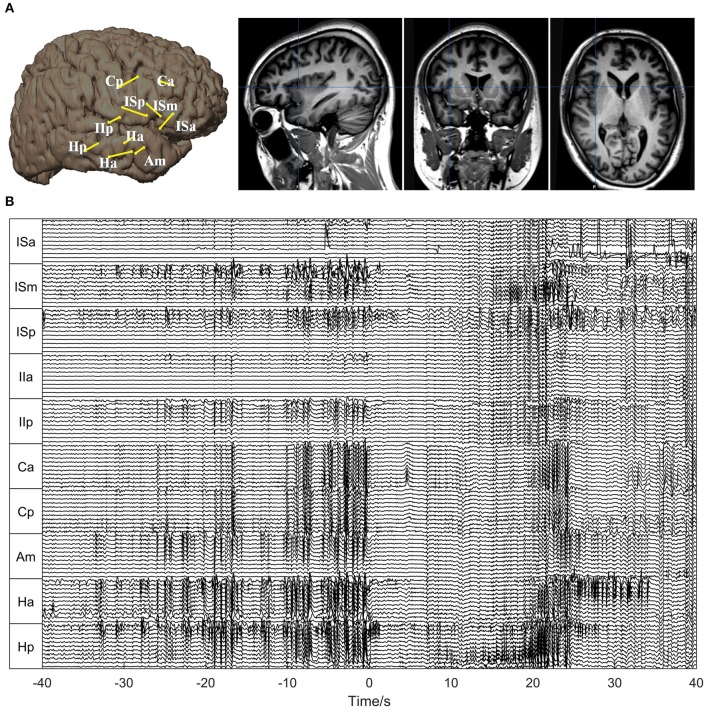
**Electrode placement and SEEG signals for Patient 1**. **(A)** Electrode placement. The left panel shows the electrode trajectories on the 3D brain schemes. The right panel shows the trajectory of the electrode Ha superimposed on the sagittal, coronal, and axial MRI views, respectively. **(B)** The SEEG signals during one seizure. The seizure onset time is set as 0. As can be seen, the seizure started with typical low-amplitude fast activities, preceded by interictal (pre-ictal) spikes.

For Patient 1, 10 depth electrodes were stereotactically implanted into the hippocampus (Ha, Hp), insula (ISa, ISm, ISp, IIa, IIp), cingulate gyrus (Ca, Cp), and amygdala (Am) (Figure 3A). Each electrode had 12 contacts, numbered from the inside out as 01, 02, …, and 12, and the outermost channel numbered 12 was removed because it was close to the skull. The SEEG signals used for the analysis are shown in Figure 3B.

**Figure 4:**
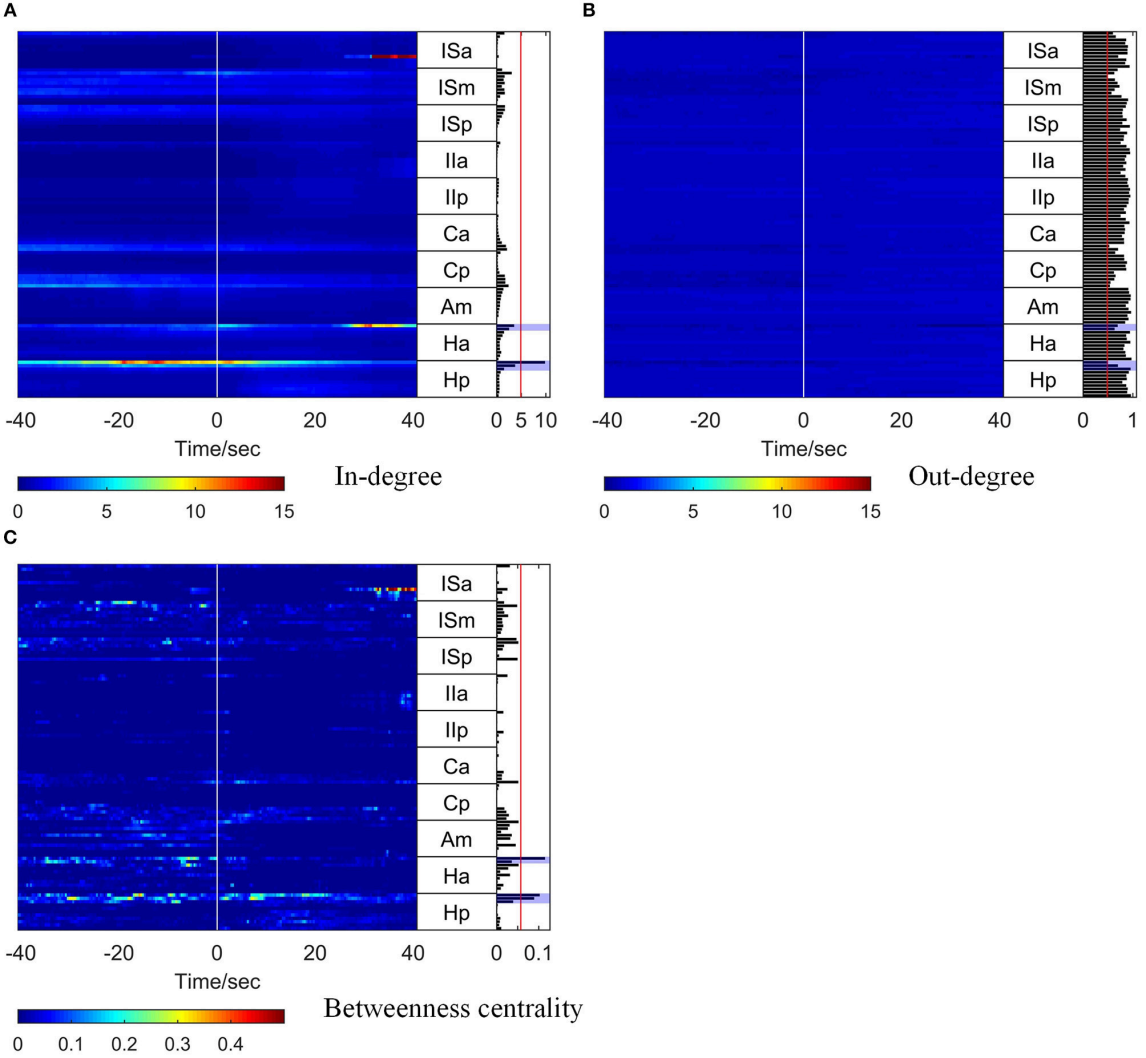
**Graph analysis results for Patient 1**. For each subfigure, the left panel shows the in-degree **(A)**, out-degree **(B)**, and betweenness centrality **(C)**. The middle panel indicates the names of the SEEG electrodes. The right panel shows the mean values of the in-degree **(A)**, out-degree **(B)**, and betweenness centrality **(C)** within a window from 10 s before to 5 s after seizure onset. The brain regions where the contacts with values exceeding the threshold (50% of maximum, red line) were located were considered to be the identified EZs. The clinically identified EZs are marked by violet shading.

The corresponding time-varying graph indices (in-degree, out-degree, and betweenness centrality) are shown in Figure 4. In order to compare the in-degree and out-degree, the scales of the color bars are set to be equal. Around the seizure onset time, Hp 01 and Hp 02 showed high in-degree values. The in-degree of Hp 01 retained a high value for quite a long time in the pre-ictal period and increased remarkably at about 20 s before seizure onset. The in-degree of Ha 01 increased at seizure onset and sustained this value for 5 s, then gradually decreased and increased again together with ISa 08 at about 25 s after seizure onset. For the betweenness centrality, the values of Hp 01, Hp 02, and Ha 01 also increased before seizure onset, and the value of ISa 08 increased remarkably at 25 s after seizure onset. These results indicate that the network connection had already changed before seizure onset and that Hp 01, Hp 02, and Ha 01 played a key role in the seizure generation. Moreover, ISa 08 may have played an important role in seizure propagation. However, the out-degree did not reveal any useful information, because the values were almost the same for all the electrode contacts for the whole period studied.

To obtain a comprehensive result for identification of EZs, the mean values of each graph index within a 15 s window (10 s before and 5 s after seizure onset) were calculated, and the results are shown in the right panel of each subfigure in Figure 4.

To identify the EZs, the brain regions where the electrode contacts with values exceeding a threshold (50% of the maximum) were located (the red line in the right panel of each subfigure in Figure 4) were considered as the identified EZs. For Patient 1, the EZs identified by clinical epileptologists were Ha 01, Hp 01, and Hp 02, which are indicated by violet shading in Figure 4. The in-degree index identified Hp 01 and the betweenness centrality identified Ha 01, Hp 01, and Hp 02, which were located in the EZs identified clinically. However, the out-degree index did not indicate any EZs.

**Figure 5:**
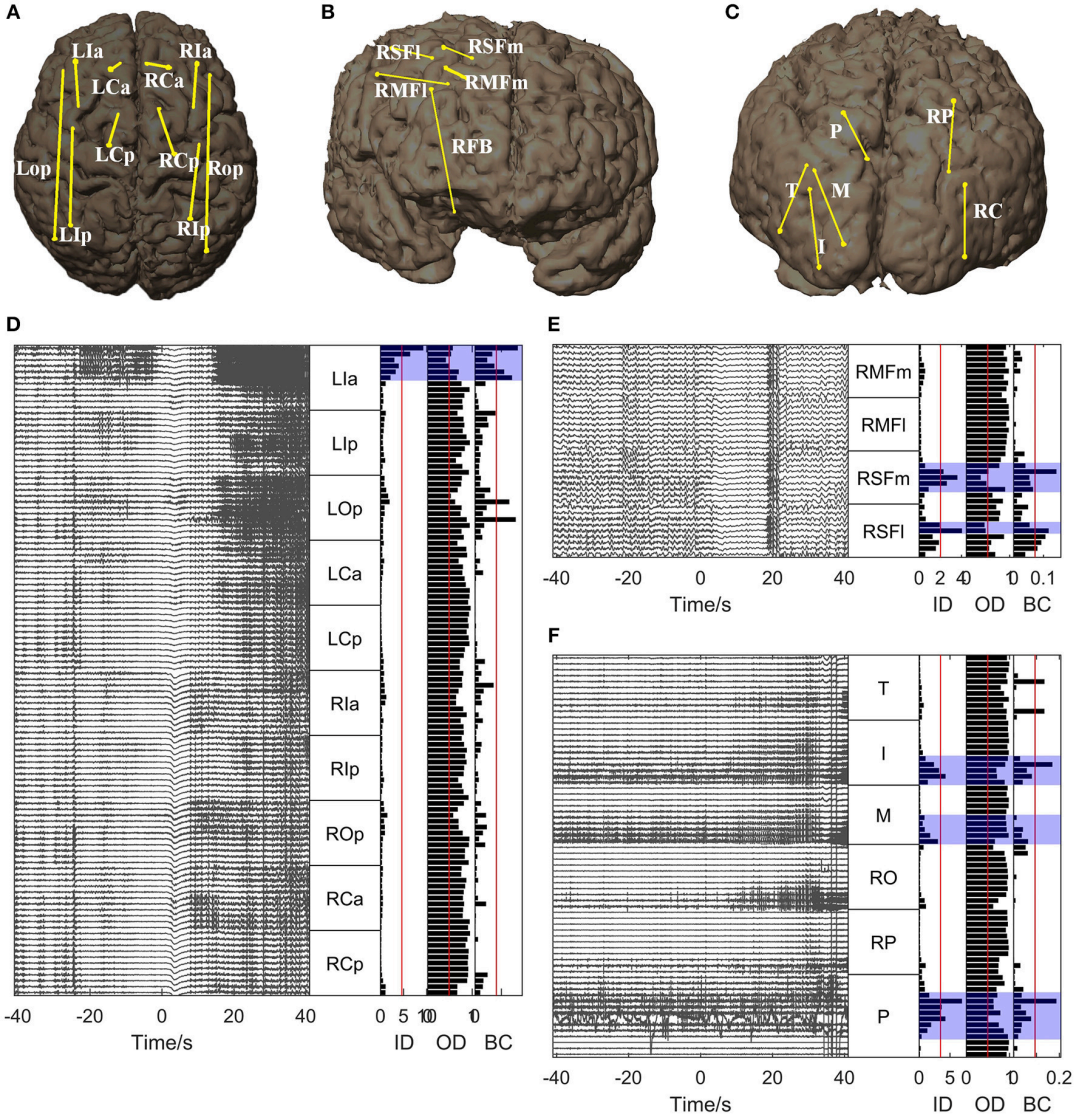
**Graph analysis results for Patients 2, 3, and 4**. **(A–C)** Show the placement of SEEG electrodes for Patients 2, 3, and 4, respectively. **(D–F)** Show the graph analysis results for Patients 2, 3, and 4 respectively. For each subfigure **(D–F)**, the left panel shows the SEEG signals during one seizure, and the right panel shows the mean values of the in-degree (ID), out-degree (OD), and betweenness centrality (BC) within a window from 10 s before to 5 s after seizure onset. The brain regions where the contacts with values exceeding the threshold (50% of maximum, red line) were located were considered to be the identified EZs. The clinically identified EZs are marked by violet shading.

**Figure 6:**
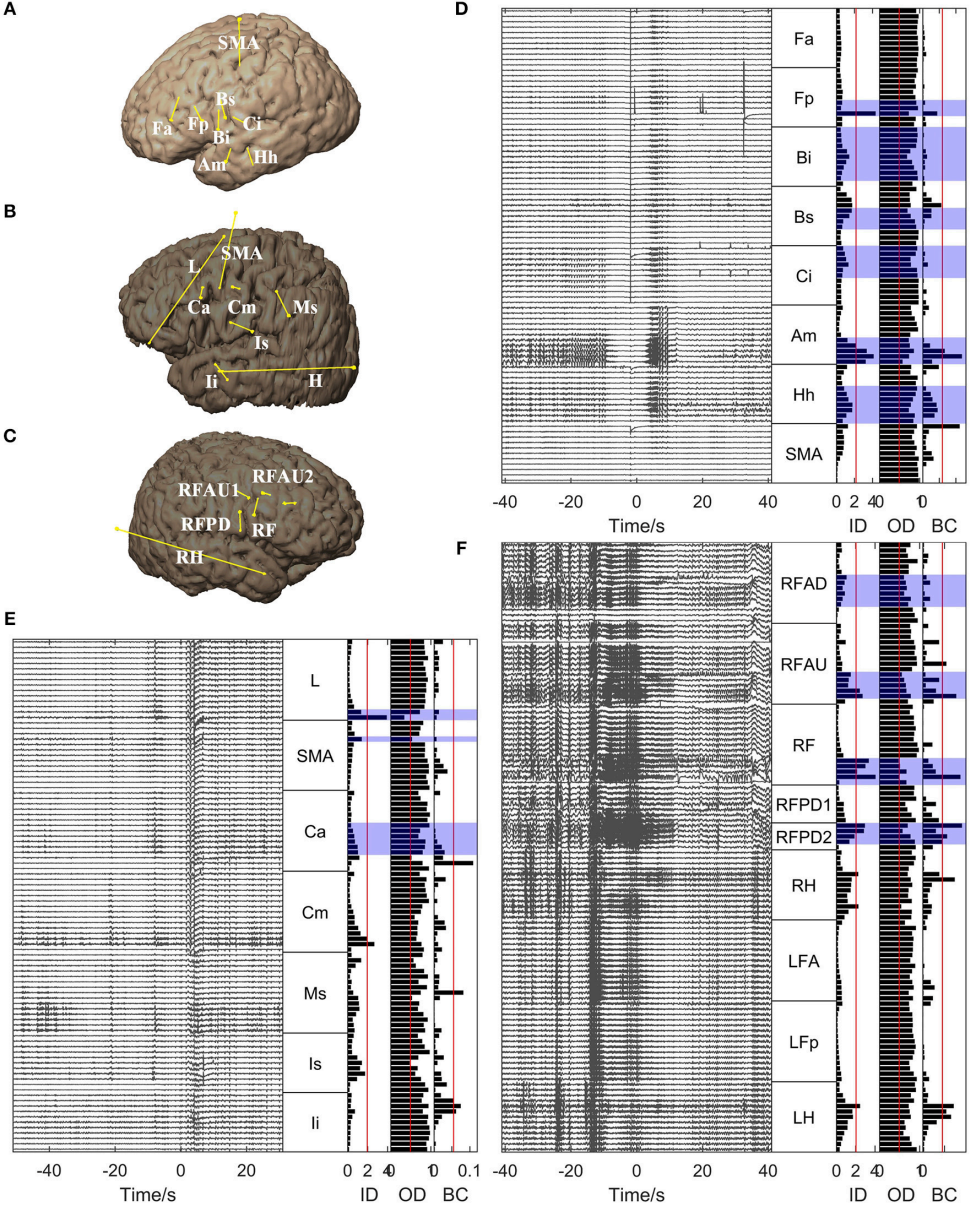
**Graph analysis results for Patients 5, 6, and 7**. **(A–C)** Show the placement of SEEG electrodes for Patients 5, 6, and 7, respectively. **(D–F)** Show the graph analysis results for Patients 5, 6, and 7 respectively. For each subfigure in **(D–F)**, the left panel shows the SEEG signals during one seizure, and the right panel shows the mean values of the in-degree (ID), out-degree (OD), and betweenness centrality (BC) within a window from 10 s before to 5 s after seizure onset. The brain regions where the contacts with values exceeding the threshold (50% of maximum, red line) were located were considered to be the identified EZs. The clinically identified EZs are marked by violet shading.

For all the other patients, the brain regions where the electrode contacts showing high in-degree and betweenness centrality values were located coincided closely with the EZs identified clinically (Figures 5, 6). However, the out-degree did not indicate any EZs for any of the patients.

**Figure 7:**
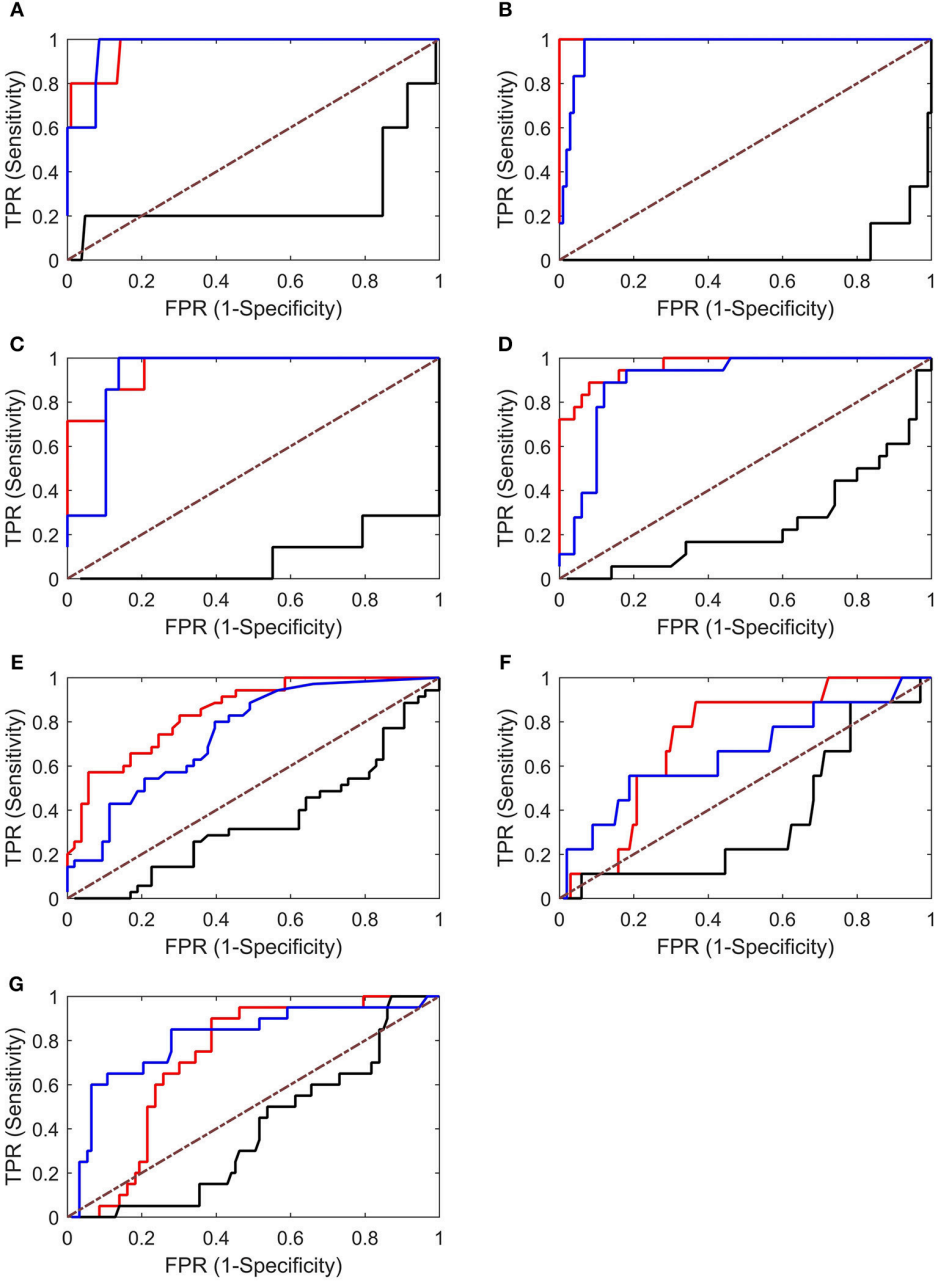
**ROC analysis results for Patients 1–7 (A–G)**. Both the in-degree (red lines) and betweenness centrality (blue lines) showed good results, whereas the out-degree (black lines) showed poor results (below the diagonal). TPR, true-positive rate; FPR, false-positive rate.

An ROC analysis was then performed, which allowed us to evaluate the ability of each graph index to identify EZs. As shown in Figure 7, the in-degree and betweenness centrality worked well in identifying EZs (with AUC values near 1). However, the values of the out-degree are all below the diagonal, indicating that the out-degree was not appropriate for identifying EZs in our cases.

## 4. Discussion

In this study, we examined the applicability of a method based on time-variant effective connectivity and graph analysis to identify EZs based on SEEG signals of patients with focal refractory epilepsy. The SEEG dataset was obtained from seven patients who had undergone presurgical evaluation and for whom subsequent resective surgery gave a good outcome. By modeling the SEEG signals around seizure onset time with the TVMVAR model, the TVPDC was calculated to construct dynamic networks. Three graph indices, namely, in-degree, out-degree, and betweenness centrality, were calculated to identify EZs. The effectiveness of each graph index was evaluated by comparing the indicative EZs with clinical diagnoses obtained by epileptologists.

In all patients who had successful surgical outcomes, the abilities of the three graph indices to identify the EZs were different. Specifically, we found that the in-degree was most effective, but the out-degree could not identify EZs at all. This is inconsistent with previous studies suggesting that the out-degree is more suitable than the in-degree for identifying EZs (Wilke et al., 2009; Varotto et al., 2012; van Mierlo et al., 2013). The major reason for these conflicting conclusions might lie in the clinical cases that we used. To confirm this, further in-depth research is required. An ideal solution, which will be a future direction for our research, is to collect a large number of clinical cases and perform an analysis in which cases are categorized according to seizure type. Another issue is our use of the PDC rather than the DTF used in the previous studies: the PDC is more suitable for in-degree cases, whereas the DTF is more suitable for out-degree cases (Plomp et al., 2014). Although both DTF and PDC are derived from Granger causality (the MVAR or TVMVAR model coefficients), the normalization procedures are quite different. For column-wise normalized PDC (Baccalá and Sameshima, 2001), the sum of outflows is bounded by 1 for each node, which makes it insensitive to the out-degree since the latter becomes close to 1 if there are tens of nodes in a network (as shown in the right panel of Figure 4B). The same consideration applies for the in-degree calculated from the row-wise normalized DTF. In fact, we had tried both PDC and DTF, and all results indicated that the in-degree calculated from the column-wise normalized PDC matched the clinically diagnosed results best.

The betweenness centrality was also effective in identifying EZs, which is in line with the results of previous studies (Wilke et al., 2011; Varotto et al., 2012; Burns et al., 2014). Unlike the in-degree (or out-degree), which quantify the information that flows into (or out from) a node, the betweenness centrality quantifies information transition through a node. Although the degree measures and the betweenness centrality capture different aspects of connectivity, our results showed that the in-degree and betweenness centrality identified almost the same EZs. These results indicated that most of the connections in the network ended within the identified EZs (flow into) and also passed through the other identified EZs. So the EZs are not isolated, and interactions between the nodes may lead to the onset of seizures. Thus, for complicated cases in which there may be several EZs and in which these are difficult to identify, combining the betweenness centrality with the in-degree will provide more information about the network and could be useful in identifying the EZs.

The successful estimation of Granger causality (TVPDC in our case) depends on the success of the TVMVAR model, since all the necessary information is derived from the model coefficients. In practice, the model order is an important issue. If the model order is too low, the model will not capture the essential dynamics of the dataset. If the model order is too high, it will also capture unwanted components, leading to overfitting and instability. For the traditional MVAR model, the model order can be optimized by criteria like the Akaike information criterion (AIC) (Akaike, 1974) or the Bayesian information criterion (BIC) (Schwarz, 1978), both of which take the measurement noise covariance matrix into account. However, for non-stationary time series, it may be not accurate to estimate the model order by applying these criteria (Havlicek et al., 2010). In this study, we tried different model orders, including 3, 5, 7, 9, and 11. We found that if the model order was too small (i.e., 3 or 5), the results had very little similarity to the clinical diagnosis. For model orders larger than 7, we obtained the same results as for 7. So we set the TVMVAR model order as 7, similarly to a study with similar conditions and settings (van Mierlo et al., 2011a, 2013). This setting gave us acceptable computational requirements and sufficient precision in the results.

In this study, the adaptation process of the Kalman filter was first evaluated in a quantitative manner. As far as we know, this is the first report of such an evaluation in any of the relevant studies. With this evaluation, the adaptation time of the Kalman filter could also be determined and the update coefficients (λ and Λ) could be properly selected. Generally, the update coefficients determine the adaptation speed. The larger the update coefficients, the more rapidly the Kalman filter follows changes in the dataset. However, a larger update coefficient may lead the filter to become unstable. van Mierlo et al. (2011a) chose the update coefficient as 10^−3^ based on knowledge gained from a previous simulation study and discarded the coefficient matrices of the TVMVAR model from the first 5 s during the adaptation. In our study, the optimal update coefficients were the same as theirs, but the adaptation time was quite different (62 ± 6.68 s, mean ± SD, *n* = 7). Actually, the states estimated during the adaptation process were influenced by the selected start time of the Kalman filter, and the results are incorrect and should be discarded. To precisely identify EZs, it is necessary to ensure correct construction of the network around the seizure onset time. Thus, the adaptation time should be precisely estimated, and for this the evaluation of the adaptation of the Kalman filter is indispensable.

During identification of the EZs, we calculated the mean values of the graph indices within a 15 s window around the seizure onset time (10 s before and 5 s after). Other studies used different windows, either crossing the seizure onset time with a few seconds before and after (Guye et al., 2006; van Mierlo et al., 2011b) or starting from the seizure onset time with a length ranging from a few seconds to 20 s (Wilke et al., 2010; David et al., 2011; Lu et al., 2012). The underlying network characteristics are believed to change before seizure onset. Although the exact time point at which this change occurs is not clear, the network should contain valuable information on seizure generation within a few seconds before onset. On the other hand, the seizure may rapidly propagate from the seizure onset zones to other brain regions within a few seconds or even more rapidly. As the EZs are the brain regions involved in the generation and early propagation of seizures, the window should be located within the seizure generation and early propagation phase. Thus, the time period during which the seizure has already become widespread has not been included in the window.

We chose the threshold used in identifying the EZs as 50% of the maximum (Wilke et al., 2009, 2011). This choice of threshold is acceptable since it filters out most of the outstanding regions with relatively high graph index values. In clinical settings, epileptologists identify EZs from information collected in multiple ways. So, for them, this rough selection of a threshold is sufficient, because all the information provided by this framework marks out the outstanding regions around seizure onset. Epileptologists may then comprehensively analyze the results together with other clinical indices to identify EZs.

Because the spatial sampling of the SEEG recordings is discrete and limited, if the SEEG electrodes do not cover the real EZs, the framework may produce erroneous results. Therefore, care should be taken when interpreting results calculated from SEEG recordings. Burns et al. (2014) found that the inadequate coverage of the EZs could lead to inconsistent brain states. We analyzed several seizures for each patient (data not shown) and obtained similar results. Therefore, it can be confirmed that the electrode coverage was adequate for the clinical cases we studied.

In summary, the applicability of an effective connectivity and graph theory analysis to localize EZs from SEEG recordings around seizure onset time was investigated in seven patients. We found that the EZs defined by our method corresponded well to the results of clinical diagnosis performed by epileptologists. Furthermore, among the three graph measures investigated, the in-degree is the best at identifying EZs. The betweenness centrality can reveal more information about the epileptic network and can also be used to identity EZs, but the out-degree seems to be unable to identify any of the EZs in our cases.

## Author contributions

JM, XY, YL, PZ, and JX conceived and designed the experiments. JM, XY, and JX performed the experiments. JM, YL, PZ, and PL analyzed the data. JM, YL, and PZ contributed materials and analysis tools. JM, PZ, YL, XY, PL, and JX wrote the paper.

### Conflict of interest statement

The authors declare that the research was conducted in the absence of any commercial or financial relationships that could be construed as a potential conflict of interest.
